# The Development and Evaluation of ‘Farm Animal Welfare’: An Educational Computer Game for Children

**DOI:** 10.3390/ani9030091

**Published:** 2019-03-13

**Authors:** Roxanne D. Hawkins, Gilly A. R. Mendes Ferreira, Joanne M. Williams

**Affiliations:** 1 Psychology, School of Media, Culture and Society, University of West Scotland, Paisley PA1 2BE, UK; 2 Scottish SPCA, Kingseat Road, Halbeath, Dunfermline, Fife KY11 8RY, UK; gilly.ferreira@scottishspca.org; 3 Clinical and Health Psychology, University of Edinburgh, Edinburgh EH8 9AG, UK; jo.williams@ed.ac.uk

**Keywords:** children, farm animals, animal welfare, education, technology

## Abstract

**Simple Summary:**

The aim of this study was to design and evaluate a new digital game ‘Farm Animal Welfare’ to teach children about farm animal welfare. The game focuses on chickens and cows, and children played the game on touchscreen netbooks. To evaluate the game, we measured children’s knowledge, attitudes, compassion, and beliefs about whether farm animals have emotions and feelings, both before and after the game, using a child-friendly questionnaire. We found that the new game led to increases in children’s knowledge about animal welfare, knowledge about welfare in different farming systems (such as caged hens vs. free range), and children were more likely to believe that farm animals can feel emotions. The game did not seem to impact children’s attitudes about cruelty or compassion towards farm animals. The new game shows promise, and to improve children’s understanding of animal welfare, we recommend further research on digital animal welfare education interventions for children.

**Abstract:**

Many children growing up in urban areas of Western countries have limited contact with and knowledge of farm animals and food production systems. Education can play an important role in children’s understanding of farm animal welfare issues, however, most education provided focuses on pets. There is a need to develop new farm animal welfare interventions for young children. This study examines the process of designing, developing, and evaluating the effectiveness of a new theoretically-driven digital game to teach children, aged 6–13 years, about farm animal welfare. ‘Farm Animal Welfare’ aimed to promote children’s knowledge about animal welfare, promote beliefs about animal sentience, and promote positive attitudes and compassion. A quasi-experimental design was carried out, using self-report questionnaires that children (*n* = 133, test = 69, control = 64) completed in the classroom. Test and control groups were from different schools and the control group did not engage in the intervention. Findings indicate a positive impact on beliefs about animal minds, knowledge about animal welfare needs, and knowledge about welfare in different farming systems, but there was no change in compassion or attitudes about cruelty. This study presents the first evaluation of a digital animal welfare education intervention for children, demonstrating the benefits of incorporating ‘serious games’ into farm animal welfare education. The findings will inform future practice around farm animal welfare education interventions for primary school children.

## 1. Introduction

Public concern over the treatment of farm animals has increased over time, and production systems have faced public scrutiny, with natural living and humane treatment being central to public perceptions on what is considered good welfare [1]. However, the public lack sufficient knowledge about farm animal welfare, and often their concerns are misinformed [2]. Growing research into farm animal sentience (i.e., cognitive and emotional abilities) might be contributing to this increased moral concern over farm animal treatment, and the ‘use of animals’ [3,4], and might, in part, be contributing to the rise in vegetarians and vegans in European countries [5]. Surprisingly little research has examined children’s knowledge of farm animal welfare needs and production systems, and their attitudes towards farm animals. It has been suggested by past studies that urban children are disconnected from rural life and agricultural systems and have low levels of food knowledge [6,7]. Yet, these cognitive and attitudinal factors can underpin food and consumer choices and impact farm animal welfare. Therefore, promoting positive orientations to farm animals and informed consumer choices, from an early age, might be beneficial [7,8]. Efforts are required to encourage children in developing a duty of care for farm animals [8] and to encourage children to value positive farm animal welfare, by preventing harm, minimizing suffering, and ensuring that farm animals ‘have a good life’ [9]. The UK Government has made recommendations about farm animal welfare for children [7], emphasizing the importance of school-based education interventions to inform children as future consumers, and impact upon future animal welfare standards and practices [7,9].

Targeting younger children for animal welfare education is common amongst animal welfare organizations [10]. This is because this age range is an important time for the development of empathy, morality, and receptivity to learning about animals, leading to conceptual changes in knowledge of biology [11,12,13]. We know from previous research that children rate farm animals lower on their ability to feel emotions and display intelligence, compared to other animals such as dogs and cats, which could impact upon children’s compassion and attitudes towards farm animals [14]. Targeting children’s beliefs about farm animal minds, as well as their knowledge of farm animal welfare needs, and the welfare implications of different food production systems are essential for educational programs.

Very little research currently exists on the benefits or effectiveness of farm animal welfare education for children. Most studies of farm welfare education have focused on training for veterinarians, and very little has examined education for children. Jamieson and colleagues [8] investigated the impact of a one-off poultry welfare education event and found positive but short-term improvements in children’s knowledge and positive behavior towards poultry, and consideration of welfare needs, but not on the value afforded to animal life. In a previous study [10], we found that a one-hour educational workshop in school classes, focusing on farm animal welfare, significantly increased children’s knowledge about farm animal welfare needs and increased children’s beliefs about animal minds, which are related to compassion towards animals and acceptance of animal cruelty [14]. In-classroom animal welfare education (not focusing on farm animals specifically) has been shown to be effective for improving children’s knowledge about animals, attitudes, and empathy towards animals [15,16,17]. However, the number of studies remains insufficient to make necessary assertions about the effectiveness of classroom-based education programs for improving children’s knowledge about and attitudes towards farm animals. Written materials and in-class presentations can be valuable methods of animal welfare education [17] but more interactive and modern methods, such as the use of digital gaming interventions, might lead to a more effective and significant change [18]. 

There is an opportunity to utilize children’s interest in technology to help them connect or re-connect with the natural world, and teach them about farm animals and agriculture [19]. ‘Serious games’ are increasingly being used by environmentalists, such as ‘conservation games’ to promote conservation attitudes, through interactions with virtual conservation landscapes [19,20,21]. There is scope to use these educational advances within animal welfare education, especially in relation to animals with which children have less direct contact, such as farm animals. Gamification of education can bring about a host of benefits by encompassing the ‘key components’ of effective learning [22], such as building upon the ‘Science of Learning’ or ‘the pillars of learning’ [23,24], which are important for stimulating learning and achieving learning outcomes. Moreover, ‘serious games’ can be more effective than conventional methods at promoting children’s intrinsic motivation to learn, promoting ‘active’ learning, as well as facilitating ‘stealth learning’ (children do not realize they are learning embedded content for an enjoyable experience) [25]. The purpose of the present study was, therefore, to design and evaluate a digital educational game, focusing on farm animal welfare, to promote knowledge of and positive attitudes towards farm animals.

This study aimed to answer the following research question: Does the Farm Animal Welfare educational digital game have a significant impact on children’s beliefs about farm animal minds, knowledge about farm animal welfare, compassion towards animals, and attitudes toward farm animal cruelty? It was hypothesized that there would be a significant pre- to post-test change for all target outcomes for the intervention group, but not the control group. The present study also took into consideration two factors that have been found to affect the baseline scores relating to children’s knowledge and attitudes towards animals: gender and age [26,27].

## 2. Materials and Methods

### 2.1. The Development of ‘Farm Animal Welfare’ Digital Game

Farm Animal Welfare is an educational or ‘serious game’ designed to teach primary school children about farm animal welfare. A series of three interactive levels were developed for each of two types of farm animals (cows and chickens), incorporating text, images, and sound. Children received feedback throughout the game and viewed their scores. All images were purchased from photo stock websites. Once developed, the game was downloaded and played offline, via touchscreen notebook computers, in a school classroom. See Supplementary Materials (Figure S1) for example screenshots of the game.

**Figure 1:**
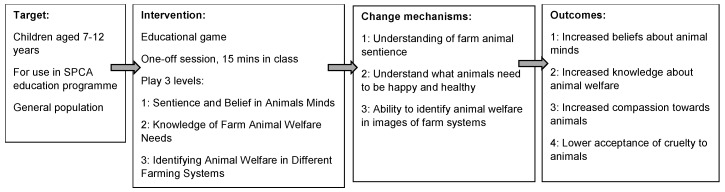
Logic Model for Farm Animal Welfare game.

All content and feedback provided within the game was based on current scientific research and confirmed by animal welfare experts to ensure accuracy and to avoid misinformation. Images were checked by animal behavior experts to ensure they accurately reflected the emotional and behavioral states they were representing in the game. Children received feedback after answering each question and gained points throughout the game for correct answers. There were three levels in the game for each of the two animals (Figure 1):

Level 1—Sentience and Belief in Animals Minds: Level 1 targeted children’s beliefs about farm animal minds. The aim of this level was to teach children that farm animals are sentient and to change their beliefs about farm animal minds. Children were provided with photographic images of animals in different emotional and welfare states (positive and negative) and were asked sentience questions relating to each image (e.g., “Is this chicken in pain?”), which the children would rate on a scale (‘not at all’, ‘not really’, ‘maybe’, ‘yes’, ‘yes very’). The questions (see Supplementary Materials Table S1) focused on the items from the Children’s Beliefs about Animal Minds measure, a key target variable, due to concerns highlighted in previous research about children’s low beliefs in farm animal minds [14].

Level 2—Knowledge of Farm Animal Welfare Needs: Level 2 aimed to promote new knowledge of the welfare needs of farm animals, focusing on the five welfare needs/freedoms. This level focused on what farm animals need to be ‘happy and healthy’ through a ‘drag and drop’ game. For each animal, children had options of items (e.g., straw) and distractors (e.g., dog lead) to move on screen. Children were required to drag and drop items that were required for animal welfare to the target animal icon and distractor items to a bin icon. Incorrect answers ‘bounced back’ and so children had to keep trying until all items were on the correct location. Once finished, feedback was provided about the five welfare needs for each animal, to reinforce learning and provide further information on welfare needs.

Level 3—Identifying Animal Welfare in Different Farming Systems: Level 3 aimed to promote children’s understanding of animal welfare in different farming systems, focusing on the balance between profit (through intensive systems) and positive welfare. This level also involved a ‘drag and drop’ game, where children had to move images onto an image of a balance-scale, depending on what they judged it was better for—‘money’ or ‘happiness’—in the farming system. Once finished, feedback was provided to reinforce learning and provide context to the items.

### 2.2. Evaluation Method

#### 2.2.1. Participants

Participants were 133 primary school children, 69 test and 64 control (53% boys, 47% girls) from three schools in West Lothian, Scotland, UK. Two schools were included in the test group, and one school was in the control group. Randomization was not possible for this study, so a quasi-experimental design was used. Children were aged between 6 and 13 years (M = 9.4, SD = 1.2) and were from two age classes, 6–9 years (42%) and 10–12 years (58%). The control group was age-matched to the test group.

#### 2.2.2. Design

A mixed factorial design was used to evaluate the intervention. One variable was phase of testing (time), a repeated-measures variable with two conditions—pre-tests (day before intervention) and post-tests (two days after intervention). The between-subjects variable was the intervention condition (intervention vs. control).

#### 2.2.3. Ethical Considerations

The ethical guidelines of the British Psychological Society, specifically relating to research with children, were adopted for this research, and ethical consent was granted by the University of Edinburgh Clinical and Health Psychology Ethics Committee. All information was treated confidentially and kept in a secure location at all times; child and school data were anonymized during data preparation by adopting identity numbers.

#### 2.2.4. Intervention Materials and Procedure

The pre-tests and post-tests were conducted during three school days within one week. Children completed the pre-test on day one (Monday), played the game on day two (Tuesday), and completed the post-test on day four (Thursday). The control group followed the same pattern but did not play the game until immediately after completing the post-test questionnaire on the Thursday. The control group received their usual classes on the Tuesday, when the intervention group was engaging with the educational game. During the intervention, children took turns to play the game on the touch screen netbook computers provided by the research team. The two netbooks were set up in a quiet space within the school and children took turns throughout the day to play the game in pairs on a netbook. The researcher called two children at a time to come over from their classes to play the game on a netbook, while the rest of the class carried on with their usual school activities. The game took each child approximately 15 min to complete. A self-complete questionnaire was developed as the evaluation tool (see below). This was administered to children during class, as a pre-test and post-test. The questionnaire took each child approximately 20 min to complete and children could ask the researcher or their teacher for assistance but not for the answers. Each child received a gift as a thank you for participation (certificate and Scottish SPCA magnets and stickers). The school also received a gift for participation, including a sponsored space at a Scottish SPCA animal rescue center. 

#### 2.2.5. Pre-Test Questionnaires and Post-Test Questionnaires

A short, child-friendly, paper questionnaire served as the evaluation tool for this study. The questionnaire used appropriate language and terminology for 6- to 13-year-olds and was piloted before the study. The questionnaire included questions regarding age, gender, and school class, and the following measures.

Children’s Compassion towards Animals (CCA): The Children’s Compassion towards Animals measure [14] uses a 5-item scale asking the question, “What do you think about animals?” with five statements (e.g., “When I see an animal that is hurt or upset I feel upset” and “When I see an animal that is hurt or upset I want to help it”). The measure was scored on a 5-point Likert scale (“Strongly disagree” to “Strongly agree”). Total scores were calculated (range 5–25). This measure demonstrated good reliability within the current sample (α = 0.71).

Children’s Beliefs about Farm Animal Minds: An adapted version of the Children’s Beliefs about Animal Minds measure [14] was created for the purpose of this evaluation, changing the animal types to focus on chickens and cows. Each scale (e.g., “Do you think the following animals are …?”) relates to a specific sentience item (clever/pain/happiness/sadness/fear). Each item is scored on a 5-point Likert scale (“Strongly disagree” to “Strongly agree”). Total scores were calculated for each animal (score range 5–25), as well as an overall beliefs about farm animal minds (BAM) score, across the two animal types (score range 10–50). The measure demonstrated a high reliability within the current sample (α = 0.84).

Acceptance of Cruelty to Farm Animals (chickens and cows): An adapted version of the Children’s Acceptance of Cruelty to Pets [18,28] measure was created for this evaluation to focus on attitudes towards cruelty to cows and chickens. This measure included two 9-item scales, with the question, “Do you think it is alright to...?” with nine statements (e.g., “Not give a chicken a comfortable place to live?”). The measure was based on animal sentience (e.g., “Make a chicken scared?” and “injure a cow”), and welfare needs (e.g., “Get treatment for an ill or injured chicken?”). The measure comprised of two separate scales, one for each animal type. Each item was scored on a 5-point Likert scale (“Strongly disagree” to “Strongly agree”). Total scores were calculated for each animal (score range 9–45), as well as an overall cruelty attitude score across the two animals (score range 18–90), where high scores indicate a high acceptance of animal cruelty. This measure showed high reliability within the current sample (α = 0.83).

Children’s Knowledge about Farm Animals Welfare: This knowledge question asked, “What do cows/chickens need to be happy and healthy?” An image of each animal was provided with space around the image, for children to write freely. Answers were coded according to the five animal welfare needs/freedoms. For example, mentioning food and water would score two points for ‘freedom from thirst, hunger, and malnutrition’. Total scores for each animal were calculated as well as a total knowledge score across animals. The measure demonstrated very good reliability within the current sample (α = 0.70).

Knowledge about Animal Welfare in Farming Systems: Children were asked, “Which farm systems are better for animal welfare and which are better for making money?” They were presented with a series of images of different farming systems for both cows and chickens (battery farm, free range, organic, and crated), the same images as within the game. Children were asked to identify which images displayed animals in a better welfare state. The measure demonstrated very good reliability (α = 0.84).

### 2.3. Analysis

For the purpose of this evaluation, total scores were summed for each key variable for each individual at each sample point, and data were analyzed at an individual level, using the Statistical package for the Social Sciences Statistics 22 (IBM Corp. Released 2013. IBM SPSS Statistics for Windows, Version 22.0. Armonk, NY: IBM Corp.), with a two-tailed significance of *p* < 0.05. A two-way repeated measures ANOVA using time (phase of testing—pre-test and post-test) as the within-subject variable, and group (two conditions—test, control) as the between-subject variable, tested main effects and interaction effects. The main focus of the results reported here is the interaction effects, which showed a difference in performance between the intervention group and the control group.

## 3. Results

### 3.1. Compassion towards Animals

**Figure 2:**
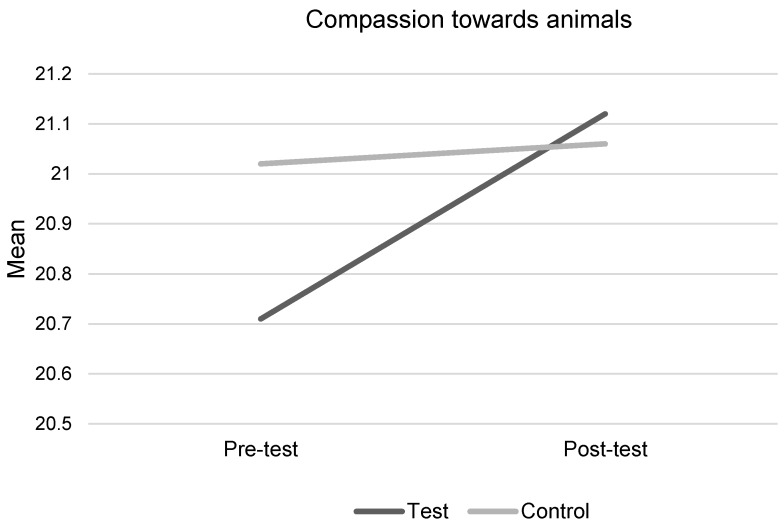
Children’s scores for compassion towards animals.

Farm Animal Welfare did not significantly improve children’s scores for compassion towards animals (Figure 2). No statistically significant interaction between the intervention condition and time was found (F(1,121) = 0.32, *p* = 0.576, η^2^ = 0.003) (Table 1 and Table 2). This result remained non-significant, when adjusting for pre-test scores and age and gender, using an analysis of covariance (ANCOVA) (F(1,123) = 0.189, *p* = 0.665, η^2^ = 0.002), even though the control group scored significantly higher than the test group, at baseline (*p* = 0.0001). Independent *t*-test at pre-test found no significant age or gender differences in compassion scores (*p* > 0.05).

### 3.2. Beliefs about Farm Animal Minds

**Figure 3:**
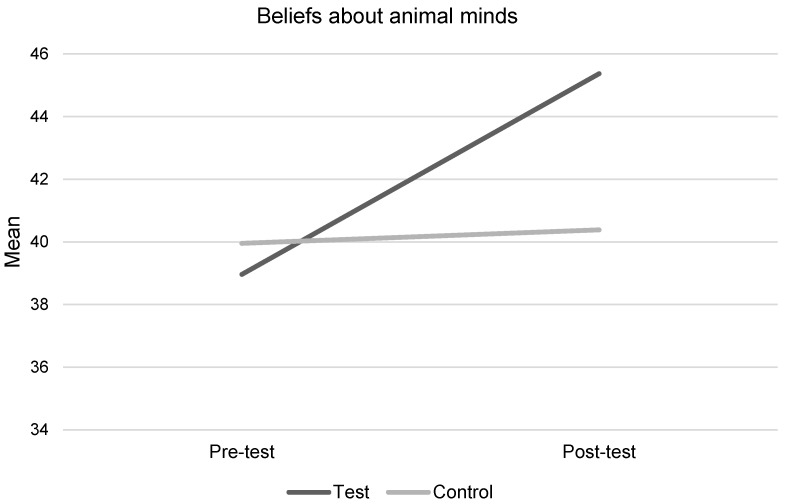
Children’s beliefs about farm animal minds.

Beliefs about farm animal minds scores improved significantly following the intervention (Table 3 and Table 4 and Figure 3). There was a statistically significant interaction between intervention condition and time (F(1,116) = 20.92, *p* = 0.0001, η^2^ = 0.15); the intervention group significantly improved at post-test, whereas the control group did not. The difference between the game intervention and control, at post-test, remained significant when adjusting for pre-test scores, age, and gender, using ANCOVA (F(1,118) = 20.79, *p* = 0.0001, η^2^ = 0.16), as the control group scored significantly higher than the test group at baseline (*p* = 0.0001). Independent *t*-test at pre-test found no significant age or gender differences in BAM scores (*p* > 0.05). 

#### 3.2.1. Beliefs about Chickens’ Minds

There was a statistically significant interaction between intervention condition and time (F(1,119) = 17.0, *p* = 0.0001, η^2^ = 0.13); the intervention group significantly improved at post-test, whereas the control group did not. The difference between game intervention and control at post-test remained significant, when adjusting for pre-test scores, age, and gender, using ANCOVA (F(1,121) = 19.5, *p* = 0.0001, η^2^ = 0.14), as the control group scored significantly higher than the test group at baseline (*p* = 0.0001). Independent *t*-tests at pre-test found no significant differences in scores by age or gender (*p* > 0.05).

#### 3.2.2. Beliefs about Cows’ Minds

There was a statistically significant interaction between intervention condition and time (F(1,117) = 17.7, *p* = 0.0001, η^2^ = 0.13); the intervention group significantly improved at post-test whereas the control group did not. The difference between game intervention and control at post-test remained significant when adjusting for pre-test scores, age, and gender, using ANCOVA (F(1,119) = 17.07, *p* = 0.0001, η^2^ = 0.13), as the control group scored significantly higher than the test group at baseline (*p* = 0.0001). Independent *t*-tests at pre-test found no significant differences in scores by age or gender (*p* > 0.05). 

### 3.3. Attitudes towards Cruelty to Farm Animals

**Figure 4:**
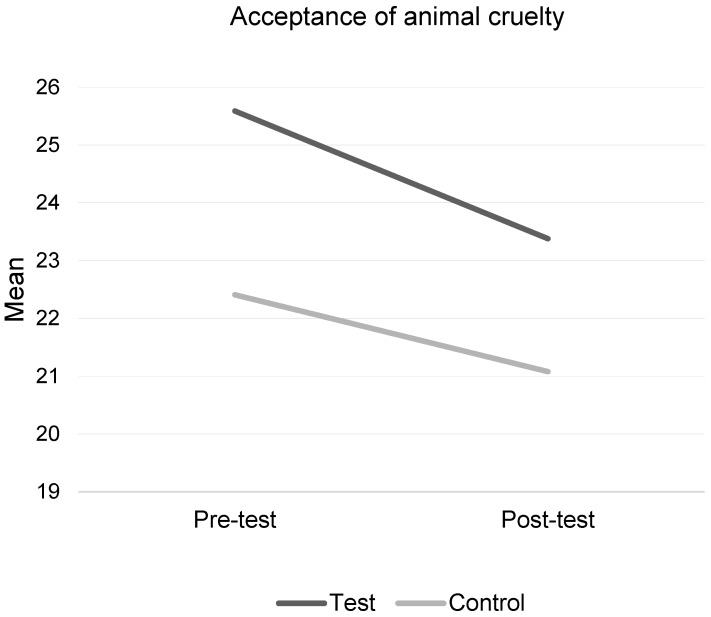
Children’s acceptance of cruelty to farm animals.

There was no significant overall change in children’s acceptance of cruelty to farm animals, following the intervention (Table 5, Figure 4). No statistically significant interaction between intervention condition and time was found (F(1,116) = 0.22, *p* = 0.641, η^2^ = 0.002). This result remained non-significant when adjusting for pre-test scores, age, and gender, using ANCOVA (F(1,118) = 0.682, *p* = 0.411, η^2^ = 0.006), even though the test group scored significantly higher than the control at baseline (*p* = 0.0001). Independent *t*-tests at pre-test found no significant differences in scores by age or gender (*p* > 0.05). 

#### 3.3.1. Attitudes towards Cruelty to Chickens

No statistically significant interaction between the intervention condition and time was found (F(1,119) = 0.21, *p* = 0.652, η^2^ = 0.002). This result remained nonsignificant when adjusting for pre-test scores, age, and gender, using ANCOVA (F(1,121) = 3.08, *p* = 0.082, η^2^ = 0.03), even though the test group scored significantly higher than the control at pre-test (*p* = 0.0001). Independent *t*-tests at pre-test found no significant differences in scores by age or gender (*p* > 0.05). 

#### 3.3.2. Attitudes towards Cruelty to Cows

No statistically significant interaction between the intervention condition and time was found (F(1,117) = 1.4, *p* = 0.239, η^2^ = 0.012). This result remained non-significant when adjusting for pre-test scores, age, and gender, using ANCOVA (F(1,119) = 0.05, *p* = 0.82, η^2^ = 0.001), even though the test group scored significantly higher than the control at pre-test (*p* = 0.001). Independent *t*-tests at pre-test found no significant differences in scores by age or gender (*p* > 0.05). 

### 3.4. Knowledge about Farm Animal Welfare Needs

**Figure 5:**
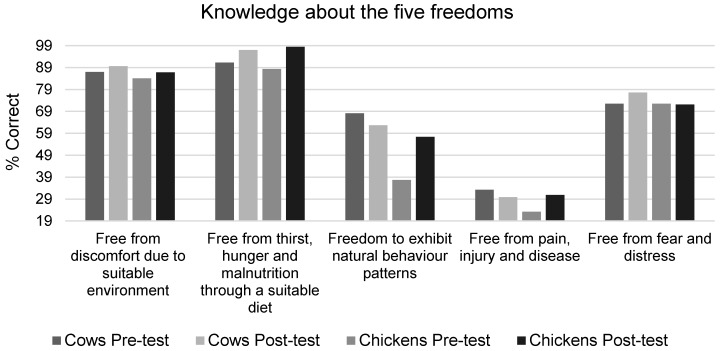
Children’s knowledge about the five freedoms for animals.

Farm Animal Welfare improved children’s scores for knowledge of welfare needs (Table 6 and Table 7 and Figure 5). No statistically significant interaction between the intervention condition and time was found (F(1,114) = 3.72, *p* = 0.056, η^2^ = 0.032), but a significant difference between the test and the control, was found at post-test, when adjusting for pre-test scores, age, and gender, using ANCOVA (F(1,116) = 26.8, *p* = 0.0001, η^2^ = 0.194), as the test group scored significantly higher than the control at baseline (*p* = 0.0001). Independent *t*-tests at pre-test found no significant differences in scores by age (*p* > 0.05), but girls scored significantly higher than boys (*t*(127) = −2.71, *p* = 0.008). 

#### 3.4.1. Knowledge of Chicken Welfare Needs

There was a statistically significant interaction between the intervention condition and time (F(1,118) = 6.15, *p* = 0.015, η^2^ = 0.05), the intervention group significantly improved at post-test, whereas the control group did not. The difference between the intervention and the control at post-test remained significant, when adjusting for pre-test scores, age, and gender, using ANCOVA (F(1,120) = 102, *p* = 0.0001, η^2^ = 0.201), as the test group scored significantly higher than the control at baseline (*p* = 0.0001). Independent *t*-tests at pre-test found no significant differences in scores by age (*p* > 0.05), but girls scored significantly higher on chicken welfare needs knowledge than boys (*t*(129) = −2.65, *p* = 0.009). 

#### 3.4.2. Knowledge of Cow Welfare Needs

No statistically significant interaction between the intervention condition and time was found (F(1,117) = 1.26, *p* = 0.264, η^2^ = 0.011), but a significant difference between the test and the control was found at post-test, when adjusting for pre-test scores, age, and gender, using ANCOVA (F(1,119) = 24.02, *p* = 0.0001, η^2^ = 0.174), as the test group scored significantly higher than the control at baseline (*p* = 0.0001). Independent *t*-tests at pre-test found no significant differences in scores by age (*p* > 0.05), but girls scored significantly higher on cow welfare needs knowledge than boys (*t*(129) = −3.02, *p* = 0.003). 

### 3.5. Knowledge of Animal Welfare in Farming Systems

**Figure 6:**
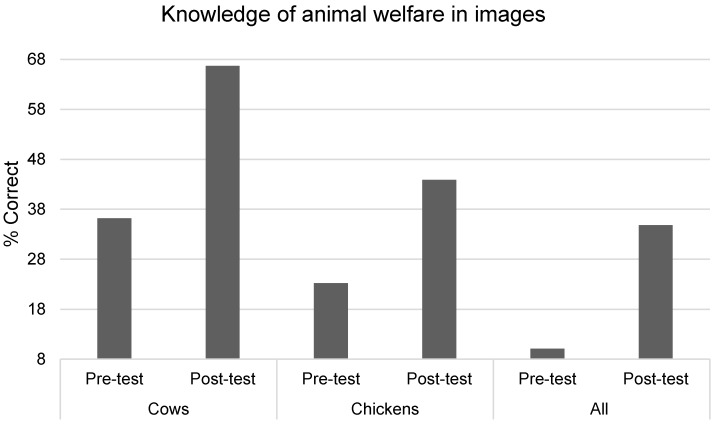
Children’s knowledge about animal welfare in farming systems.

There was a statistically significant interaction between the intervention condition and time (F(1,115) = 8.81, *p* = 0.004, η^2^ = 0.071) for overall understanding of welfare in food production systems (Table 8, Figure 6). However, the significant difference between intervention and control groups at post-test was lost, after adjusting for pre-test scores, age, and gender, using ANCOVA (F(1,117) = 3.65, *p* = 0.059, η^2^ = 0.032), as the control group scored significantly higher than the test group at baseline (*p* = 0.0001). Independent *t*-tests at pre-test found no significant differences in scores by age or gender (*p* > 0.05).

#### 3.5.1. Knowledge of Chicken Welfare and Farming Systems

There was a statistically significant interaction between the intervention condition and time (F(1,115) = 10.31, *p* = 0.002, η^2^ = 0.082); the intervention group significantly improved at post-test, whereas the control group did not. The difference between intervention and control at post-test remained significant, when adjusting for pre-test scores, age, and gender, using ANCOVA (F(1,117) = 5.33, *p* = 0.023, η^2^ = 0.05), as the test group scored significantly higher than control at baseline (*p* = 0.0001). Independent *t*-tests at pre-test found no significant differences in scores for age (*p* > 0.05), but boys scored significantly higher than girls (*t*(129) = −2.794, *p* = 0.006). 

#### 3.5.2. Knowledge of Cow Welfare and Farming Systems

There was a statistically significant interaction between the intervention condition and time (F(1,115) = 8.81, *p* = 0.004, η^2^ = 0.07); the intervention group significantly improved at post-test whereas the control group did not. The difference between intervention and control at post-test remained significant when adjusting for pre-test scores, age, and gender, using ANCOVA (F(1,117) = 5.33, *p* = 0.023, η^2^ = 0.05), as the control group scored significantly higher at pre-test (*p* = 0.000). Independent *t*-tests at pre-test found no significant differences in scores by age or gender (*p* > 0.05).

## 4. Discussion

The purpose of this study was to evaluate the effectiveness of a novel digital farm animal welfare educational intervention named ‘Farm Animal Welfare’. The game had a significant impact on children’s beliefs about farm animal minds, children’s knowledge about farm animal welfare needs, and knowledge about farm animal welfare in different farming systems. The game did not however, have an impact on children’s compassion towards farm animals or their acceptance of cruelty to farm animals. The results, therefore, only partially supported the hypotheses. 

The present study demonstrated that a one-off gaming intervention can lead to positive increases in children’s knowledge about animal welfare. This is in line with previous research on short-term animal welfare education interventions and might be because knowledge is the easiest variable to change through education [10,28]. Although no overall difference in scores for knowledge about animal welfare in farming systems was found, when analyzing the scores separately for each type of farm animal, significant improvements were found. In combination, these findings are promising, given that knowledge about animal welfare needs in school-aged children is generally low, especially for farm animals [8,28]. Although an overall significant change was found for knowledge about farm animal welfare needs, when examining the percentages of children who mentioned each welfare need/freedom, changes were only seen for some of the welfare needs/freedoms. For example, very few children mentioned freedom from pain, injury, and disease for either the cow and the chicken, and this percentage did not improve at post-test for cows, but did so for chickens. Children were more likely to mention the dietary needs of farm animals and were less likely to mention the health needs or the freedom to exhibit natural behavior patterns, this is consistent with previous findings for children’s knowledge about the welfare needs of pet animals [29]. Children were able to identify the need for farm animals to be free from fear and distress, and along with children’s scores on beliefs about animal minds and low acceptance of cruelty to farm animals, this demonstrated that children had some awareness that farm animals are sentient and can feel pain and fear, following the intervention. At pre-test, only 10% of children in the test group were able to identify animal welfare in all farming system images, compared to 36% at post-test, which is still relatively low. However, when looking at the individual animal types, children were better able to identify animal welfare in the cow farming systems, following the intervention (with 67% scoring correctly at post-test, compared to 36% for cows at post-test). These findings reinforce the need to focus on teaching children about specific welfare needs/freedoms, in more depth, in future education programs, focusing on those that children lack knowledge of.

Children scored significantly higher on beliefs about cows’ and chickens’ minds, following the intervention, compared to the control group who did not. This finding was expected, given previous evaluations showing similar impacts of animal welfare education on children’s beliefs about animal minds, for a range of animal types [10]. This finding supports previous hypotheses that such beliefs are easy to change through a simple intervention. These beliefs are a key cognitive factor influencing animal welfare [30,31,32,33,34], with low beliefs in animal minds being predicative of negative interactions child-animal interactions [33,34]. It is, therefore, a positive note that this digital game improved beliefs about farm animal minds, as these beliefs tend to be quite low at baseline (in this study, children scored 39 out of a possible 50, children rated chickens’ mental abilities slightly higher than cows), compared to other animal types [29,34]. Furthermore, farm animals can also be a target for animal cruelty and so examining ways of preventing cruelty to farm animals, starting early in childhood, is critical. 

Although children scored higher on compassion at post-test, no overall significant difference was found. Compassion scores were relatively high at baseline (mean of 20.71 out of a possible 25), which is consistent with previous studies on children’s compassion towards pets and children’s compassion towards animals in general [10,29]. No age or gender differences were found for compassion towards farm animals, which was unexpected given research on gender differences in adults’ compassion towards animals. Perhaps the sample size was too small to find a difference, or perhaps gender differences do not emerge until later in life, we cannot ascertain these potential developmental trends without an assessment of compassion across a wider age-range. It has been suggested that children might need direct contact and interaction with animals, and a chance to form a bond or attachment to an animal, to develop compassion towards animals. This could be investigated in future research, given such contact and experience might impact children’s knowledge of and attitudes towards farm animals. 

Farm Animal Welfare did not significantly reduce children’s acceptance of cruelty to farm animals, in line with previous evaluations of farm animal education workshops [8]. No age or gender differences were found. A possible reason might be that children were not accepting of cruelty at baseline (scoring a mean of 25.59 out of a possible 90, at pre-test), although these scores did reduce at post-test. Similar scores were found for chickens and cows, when analyzed separately, and children were not more accepting of cruelty to one farm animal type. Farm animals were not exempt from being targets of animal cruelty, and so education interventions should aim to prevent this from occurring, but we need to examine different educational methods. Farm Animal Welfare did not have cruelty content, which might explain why no significant change was found in attitudes towards cruelty. Other game interventions that have focused on preventing animal cruelty through specific content (e.g., accidental cruelty to pets), did find a change in children’s acceptance of cruelty to pets [26]. Future interventions that aim to prevent cruelty to farm animals could, therefore, explore how to incorporate the concept of cruelty into the intervention strategies, using child-friendly materials. 

## 5. Conclusions

This study is the first to demonstrate the benefits of utilizing digital gaming technology in animal welfare education and the use of game-based learning, or ‘serious’ games for promoting children’s beliefs about farm animal minds and knowledge of farm animal welfare. Knowledge and beliefs about animal minds are the easiest to change through a digital educational intervention, but compassion seems more resistant to change and needs further investigation. Although this study presents promising findings, longer-term evaluation is required, with a larger population across wider demographics. Long-term follow-up would be useful to examine whether changes are long-lasting. Trialing such computer games, compared with more traditional educational pedagogy with a wider range of ages, could also be beneficial. Children’s feedback on the game was extremely positive and children reported wanting more animals and more levels to play and so a more complex game could be developed in the future. This game is a novel small-scale intervention that has shown a positive impact on knowledge of welfare needs, sentience, and production systems. Future games could be developed and evaluated to reveal whether they can have a similar impact on children’s welfare knowledge of other types of animals, including pets, wildlife, and zoo animals. 

## Figures and Tables

**Table 1:** Descriptive statistics for children’s compassion towards animals.

Measure Item	Test				Control			
	Pre-Test		Post-Test		Pre-Test		Post-Test	
	M	SD	M	SD	M	SD	M	SD
When I see an animal that is hurt or upset I feel upset	4.28	0.8	4.26	0.9	4.11	0.8	4.19	0.9
When I see an animal that is hurt or upset I want to help it	4.42	0.8	4.46	0.7	4.48	0.6	4.43	0.9
When I see an animal that is hurt or upset I want to tell someone	4.54	0.6	4.55	0.7	4.58	0.5	4.52	0.9
When I see an animal that is hurt or upset I think it is my responsibility to help	3.65	1	3.62	1	4.03	0.8	4.02	1
I know what to do when I see an animal that is hurt or upset	3.83	1	4.22	0.8	3.81	0.9	3.91	1
TOTAL Compassion	20.71	3	21.12	3	21.02	3	21.06	4

Note: Scores for each item ranged from 1–5. Total compassion scores ranged from 5–25.

**Table 2:** Results from main effects analysis for each intervention, following insignificant interactions.

Main Effect of Time				Main Effect of Group			
*df*	F	*p*	η^2^	*df*	F	*p*	η^2^
Compassion towards animals							
1121	0.8	0.37	0.01	1121	0.04	0.85	0.00
Total attitudes towards animal cruelty							
1116	7.3	0.008	0.059	1116	5.87	0.017	0.048
Attitudes towards cruelty to chickens							
1119	6.36	0.013	0.051	1119	7.27	0.008	0.058
Attitudes towards cruelty to cows							
1117	3.97	0.049	0.033	1117	3.92	0.05	0.032
Total knowledge about the five freedoms							
1114	5.49	0.021	0.046	1114	36.6	0.0001	0.243
Knowledge about the five freedoms for cows							
1117	8.58	0.004	0.068	1117	36.7	0.0001	0.24

**Table 3:** Descriptive statistics for children’s beliefs about farm animal minds (BAM).

Measure Item	Test				Control			
	Pre-Test		Post-Test		Pre-Test		Post-Test	
	M	SD	M	SD	M	SD	M	SD
Chicken Clever	3.06	0.9	4.39	0.7	3.11	1	3.48	1
Chicken Pain	4.36	0.9	4.67	0.7	4.13	1	4.06	1
Chicken Happy	4	0.9	4.68	0.6	4.28	0.8	4.33	0.9
Chicken Sad	3.99	0.9	4.49	0.8	4.02	1	4.12	1
Chicken Fear	4.28	1	4.57	0.8	4.33	1	4.19	1
Total Chicken Minds	19.68	3	22.80	3	19.86	3	20.17	5
Cow Clever	3.22	1	4.43	0.8	3.33	1	3.81	2
Cow Pain	4.06	1	4.57	0.8	4.15	1	4.06	1
Cow Happy	4.10	1	4.68	0.6	4.38	0.8	4.26	1
Cow Sad	3.77	1	4.41	1	4.02	1	4.06	1
Cow Fear	4.13	1	4.41	1	4.16	1	4.11	1
Total Cow Minds	19.28	4	22.50	3	20.03	4	20.30	5
TOTAL BAM	38.96	6	45.37	5	39.95	7	40.38	10

Note: Total scores for each animal type, ranged from 5–25. Overall BAM scores across all animal types, ranged from 10–50, where a high score indicated high BAM.

**Table 4:** Results for simple effects following significant interactions.

Test × Control at Pre-Test				Test × Control at Post-Test			
*df*	F	*p*	η^2^	*df*	F	*p*	η^2^
Total beliefs about farm animal minds							
130	0.759	0.385	0.006	120	13	0.0001	0.10
Beliefs about chicken minds							
131	0.11	0.743	0.001	119	15.1	0.0001	0.113
Beliefs about cow minds							
128	1.21	0.274	0.009	119	8.69	0.004	0.07
Knowledge about the five freedoms for chickens							
129	19.86	0.0001	0.133	119	40.3	0.0001	0.253
Total knowledge about farm welfare from farming system photos							
131	0.064	0.801	0.00	119	11.58	0.001	0.10
Knowledge about chicken welfare in farming system photos							
131	1.27	0.262	0.01	119	17.1	0.0001	0.128
Knowledge about cow welfare in farming system photos							
129	1.27	0.262	0.01	117	3.99	0.048	0.033

**Table 5:** Descriptive statistics for children’s attitudes towards cruelty to farm animals.

Measure Item	Test				Control			
	Pre-Test		Post-Test		Pre-Test		Post-Test	
	M	SD	M	SD	M	SD	M	SD
Attitudes towards cruelty to chickens								
Injure a chicken	1.28	0.8	1.19	0.5	1.08	0.3	1	0
Make a chicken scared	1.23	0.4	1.17	0.4	1.11	0.4	1.04	0.2
Make a chicken angry	1.28	0.5	1.16	0.4	1.11	0.4	1.02	0.1
Make a chicken sad	1.25	0.6	1.23	0.5	1.16	0.4	1.04	0.2
Make a chicken happy (r)	1.32	0.5	1.35	0.8	1.53	1	1.31	0.7
Not give a chicken food or water	1.33	0.8	1.32	0.9	1.14	0.5	1.08	0.6
Get treatment for an ill or injured chicken (r)	1.54	0.8	1.52	0.9	1.6	1	1.48	1
Not give a chicken a comfortable place to live	1.65	1	1.38	1	1.2	0.6	1.15	0.6
Not allow a chicken to perch or dig	1.80	1	1.65	1	1.27	0.6	1.21	0.7
Total cruelty to chickens	12.67	4	11.97	4	11.16	3	10.33	2
Attitudes towards cruelty to cows								
Injure a cow	1.17	0.5	1.15	0.6	1.23	0.8	1.21	0.7
Make a cow scared	1.29	0.5	1.12	0.5	1.08	0.3	1.04	0.3
Make a cow angry	1.23	0.5	1.13	0.5	1.11	0.4	1.04	0.3
Make a cow sad	1.33	0.6	1.28	0.8	1.46	0.8	1.04	1
Make a cow happy (r)	1.57	0.9	1.19	0.4	1.15	0.7	1.45	0.6
Not give a cow food or water	1.51	0.9	1.43	1	1.15	0.7	1.11	1.4
Get treatment for an ill or injured cow (r)	1.58	0.9	1.62	1	1.72	1	1.77	1
Not give a cow a comfortable place to live	1.71	1	1.29	0.9	1.23	0.7	1.11	0.6
Not allow a cow to move around or go outside	1.54	1	1.34	1	1.16	0.7	1.09	0.6
Total cruelty to cows	12.93	4	11.54	4	11.30	3	10.87	3
Total cruelty to farm animals	25.59	7	23.38	7	22.41	6	21.08	5

Note: Scores for each item ranged from 1–5. Total cruelty scores for each animal ranged from 9–45. Overall cruelty to farm animals scores, ranged from 18–90. High scores = high acceptance of cruelty.

**Table 6:** Descriptive statistics for knowledge about the five freedoms for animals, displaying the percentage of children in the intervention group who mentioned each freedom.

Measure Item	Pre-Test %		Post-Test %	
	Yes	No	Yes	No
Cows				
Free from discomfort due to suitable environment	87	13	89.6	10.4
Free from thirst, hunger and malnutrition through a suitable diet	91.3	8.7	97	3
Freedom to exhibit natural behavior patterns	68.1	31.9	62.7	37.3
Free from pain, injury and disease	33.3	66.7	29.9	70.1
Free from fear and distress	72.5	27.5	77.6	22.4
Chickens				
Free from discomfort due to suitable environment	84.1	15.9	86.8	13.2
Free from thirst, hunger and malnutrition through a suitable diet	88.4	11.6	98.5	1.5
Freedom to exhibit natural behavior patterns	37.7	62.3	57.4	42.6
Free from pain, injury and disease	23.2	76.8	30.9	69.1
Free from fear and distress	72.5	27.5	72.1	27.9

**Table 7:** Descriptive statistics for knowledge about the five freedoms for animals, displaying mean scores for each item for the intervention group.

Measure Item	Pre-Test		Post-Test	
	M	SD	M	SD
Cows				
Free from discomfort due to suitable environment	1.86	1.3	1.9	1.3
Free from thirst, hunger and malnutrition through a suitable diet	1.88	0.78	2	0.65
Freedom to exhibit natural behavior patterns	0.94	0.98	0.73	0.67
Free from pain, injury and disease	0.46	0.76	0.39	0.65
Free from fear and distress	1.43	1.3	1.37	1.2
Chickens				
Free from discomfort due to suitable environment	1.61	1.2	1.68	1.2
Free from thirst, hunger and malnutrition through a suitable diet	1.75	0.76	1.96	0.47
Freedom to exhibit natural behavior patterns	1.06	1.1	0.90	0.96
Free from pain, injury and disease	0.32	0.65	0.38	0.65
Free from fear and distress	1.25	1.1	1.34	1.2

**Table 8:** Descriptive statistics for knowledge about animal welfare in photos of farming systems.

Measure Item	Test		Control	
	Pre-Test %	Post-Test %	Pre-Test %	Post-Test %
Cow a	85.5	95.5	93.5	92.5
Cow b	68.1	89.4	71	83
Cow c	59.4	81.8	59.7	64.2
Cow d	88.4	93.9	95.2	94.3
Cow e	78.3	97	79	77.4
Cow f	76.8	92.4	75.8	84.9
Cow g	82.6	89.4	93.5	94.3
Cow h	87	95.5	95.2	94.3
& of all cow pictures correct	36.2	66.7	48.4	52.8
Chicken a	84.1	95.5	74.2	77.4
Chicken b	79.7	89.4	90.3	83
Chicken c	53.6	57.4	43.5	43.4
Chicken d	84.1	94.1	75.8	73.6
Chicken e	76.8	94.1	77.4	73.6
Chicken f	78.3	92.6	74.2	69.8
Chicken g	81.2	85.3	90.3	90.6
Chicken h	84.1	91.2	74.2	67.9
& of all chicken pictures correct	23.2	43.9	21	20.8
All photos correct	10.1	34.8	16.1	15.1

## Supplementary Materials

The following are available online at https://www.mdpi.com/2076-2615/9/3/91/s1, Figure S1: Screenshots of Farm Animal Welfare, Table S1: Full content and functionality of Farm Animal Welfare.
