# Supplementary material for: The Development and Evaluation of ‘Farm Animal Welfare’: An Educational Computer Game for Children
Source: Animals (Basel). 2019 Mar 13;9(3):91. doi: 10.3390/ani9030091 (PMC6466369; doi:10.3390/ani9030091)
Supplement: Supplementary file 1 [file animals-09-00091-s001.pdf]

Supplementary materials: Content of Farm Animal Welfare

Table S1. Full content and functionality of Farm Animal Welfare.

CHICKENS				
**LEVEL 1: PART 1 Chicken Sentience**				
**Q**	**Image**	**Question**	**Format**	**Feedback**
1a	Chicken playing in dust bath	Is this chicken happy?	Multiple format *(not at all, not really, maybe, yes, yes very)*	This chicken is happy because it has a dust bath to play in!
1b	Chicken in pain	Is this chicken in pain?	Multiple format *(not at all, not really, maybe, yes, yes very)*	This chicken is in pain
1c	Chicken in unhealthy condition	Is this chicken sad?	Multiple format *(not at all, not really, maybe, yes, yes very)*	We can see this chicken is unhappy by the way she is lying with her head down
1d	Chicken in unhealthy condition	Is this chicken scared?	Multiple format *(not at all, not really, maybe, yes, yes very)*	This chicken is unhappy and might be scared as she has not been looked after properly
1e	Healthy chickens in good condition	Are these chickens in pain?	Multiple format *(not at all, not really, maybe, yes, yes very)*	These chickens are not in pain. These are happy and healthy chickens!
1f	Chicken in unhealthy condition	Is this chicken happy?	Multiple format *(not at all, not really, maybe, yes, yes very)*	This chicken is unhappy and unhealthy as she has not been looked after properly
1g	Chicken in unhealthy conditions in intensive farming system	Are these chickens scared?	Multiple format *(not at all, not really, maybe, yes, yes very)*	These chickens are unhappy and may be scared because there are lots of chickens living close together in one barn (these are broiler chickens)
1h	Healthy chickens in good condition with fresh water	Are these chickens sad?	Multiple format *(not at all, not really, maybe, yes, yes very)*	These chickens are happy because they have fresh water to drink!
**LEVEL 1: PART 2 Chicken sentience**				
**Q**	**Image**	**Question**	**Format**	**Feedback**

2a	Neutral chicken	Are chickens clever?	Multiple format *(not at all, not really, maybe, yes, yes very)*	Chickens are cleverer than we may think they are! Did you know chickens can do basic math?!
2b	Neutral chicken	Do chickens get frightened?	Multiple format *(not at all, not really, maybe, yes, yes very)*	Chickens can get scared and anxious! Especially when there is a fox around!
2c	Neutral chicken	Can chickens feel pain?	Multiple format *(not at all, not really, maybe, yes, yes very)*	Chickens can feel pain just like we can!
2d	Neutral chicken	Can chickens feel happy?	Multiple format *(not at all, not really, maybe, yes, yes very)*	Chickens show happiness especially when they have something fun to do such as play in a dust bath!
2e	Neutral chicken	Can chickens feel sad?	Multiple format *(not at all, not really, maybe, yes, yes very)*	Chickens will be unhappy if they do not have a nice clean place to live with friends and the right food
2f	Neutral chicken	Do chickens like to live alone?	Multiple format *(not at all, not really, maybe, yes, yes very)*	Chickens like to live with other chickens! Did you know chickens like to form bonds and friendships with others?

LEVEL 2

	Question	Format	Feedback
3a	What does a chicken need to be happy and healthy?	Drag and drop items to chicken or bin *(various farm items)*	Chickens need lots of things to make them happy and healthy such as: Constant fresh food and water, A place to rest, Space to move around and flap their wings, Fresh air, Company from other chickens, Health checks and medication if needed, A clean place to live, Safe items to reduce boredom such as a dust bath or swing, Laying hens need comfy nest boxes, Shelter to hide from scary noises and movement, Items to peck and scratch at, To be treated nicely and free from pain and fear, Perches to sleep on.
3b	What does a chicken need to be happy and healthy?	Drag and drop items to chicken or bin *(various farm items)*	

LEVEL 3

Question	Format	Feedback
4. Some chickens are raised for meat, these are called broiler chickens. Some chickens are reared for eggs, these are called laying hens. Which farming systems are better for money and which are better for happy animals? Place the farming system onto the scale!	Drag and drop items to 'happy side' of the scale or 'money side' of the scale. *Images of different chicken farming systems*	Well done! Keeping lots of chickens in one space means that more eggs or more meat can be made and farmers will make more money. However, keeping lots of chickens in one space means more unhappy chickens as chickens will have less space to move around, less objects to peck and scratch at, less space to spread their wings and less places to perch.

COWS

LEVEL 1: PART 1 Cow Sentience

Q	Image	Question	Format	Feedback

5a	Cow in healthy condition	Is this cow happy?	Multiple format *(not at all, not really, maybe, yes, yes very)*	This dairy cow is happy and healthy!
5b	Cow in pain	Is this cow in pain?	Multiple format *(not at all, not really, maybe, yes, yes very)*	This beef cow is in pain
5c	Cows in intensive farming condition	Are these cows sad?	Multiple format *(not at all, not really, maybe, yes, yes very)*	These dairy cows may be unhappy because they are inside with no grass and do not have much space to move around
5d	Cow with head stuck in ladder	Is this cow scared?	Multiple format *(not at all, not really, maybe, yes, yes very)*	This dairy cow may be scared and unhappy because she has got her head stuck in a ladder!
5e	Cow in healthy condition	Are these cows in pain?	Multiple format *(not at all, not really, maybe, yes, yes very)*	These beef cows are not in pain, they are happy and healthy as they have been looked after well!
5f	Cow in unhealthy and neglected condition	Is this cow happy?	Multiple format *(not at all, not really, maybe, yes, yes very)*	This cow is unhappy because he has been neglected and not looked after properly
5g	Cow in bad condition tied up	Is this cow scared?	Multiple format *(not at all, not really, maybe, yes, yes very)*	This meat cow is scared because he has been tied up and has no space to move around
5h	Cow in healthy condition in open field	Are these cows sad?	Multiple format *(not at all, not really, maybe, yes, yes very)*	These are happy dairy cows because they have lots of space and fresh grass to eat!

LEVEL 1: PART 2

Q	Image	Question	Format	Feedback
6a	Neutral cow	Are cows clever?	Multiple format (not at all, not really, maybe, yes, yes very)	Cows are cleverer than we may think! They can solve puzzles and enjoy finding the solution! Did you know cows also have good memories?
6b	Neutral cow	Do cows get frightened?	Multiple format (not at all, not really, maybe, yes, yes very)	Cows can become scared easily by loud noises or sudden movement
6c	Neutral cow	Can cows feel pain?	Multiple format (not at all, not really, maybe, yes, yes very)	Cows can feel pain just like we can!
6d	Neutral cow	Can cows feel happy?	Multiple format (not at all, not really, maybe, yes, yes very)	Cows can be happy! Especially when there’s nice fresh grass to eat, fresh clean water and lots of space to move around in
6e	Neutral cow	Can cows feel sad?	Multiple format (not at all, not really, maybe, yes, yes very)	Cows can feel sad, especially when their calf is taken away from them. Cows shed tears when they are sad, just like we do

LEVEL 2 COW Five Welfare Needs

	Question	Format	Feedback
7a	What does a cow need to be happy and healthy?	Drag and drop items to chicken or bin	Cows need lots of things to make them happy and healthy such as: Constant fresh food and clean, fresh water, A nice comfortable place to

7b What does a cow need to be happy and healthy?	Drag and drop items to chicken or bin	rest and lie down, Lots of space to move around and not tied up, Fresh air, Other cows to make friends with and groom, Health checks and medical treatment for illness or injury, A clean place to live, Shelter to hide from scary noises and sudden movement of objects, To be treated nicely and gently, free from pain and fear
**LEVEL 3 COWS**		
**Question**	**Format**	**Feedback**
8. Some cows are raised for meat, these are called beef cattle. Some cows are reared for milk, these are called dairy cows. Which farming systems are better for money and which are better for happy animals? Place the farming system onto the scale!	Drag and drop items to 'happy side' of the scale or 'money side' of the scale. Images of different cow farming systems	Well done! Keeping lots of cows in one space means that more beef or more milk can be made and farmers will make more money. However, keeping lots of cows in one space means more unhappy cows as they will have less space to move around. Using special machines to milk dairy cows means farmers can produce more milk quickly, but this may be uncomfortable for the cows. Calves are taken away from their mothers at a very young age so that the mothers produce more, good quality milk for the farmer. Therefore the farmer makes more money but this makes the cows unhappy.

Supplementary materials: Supplementary Figures

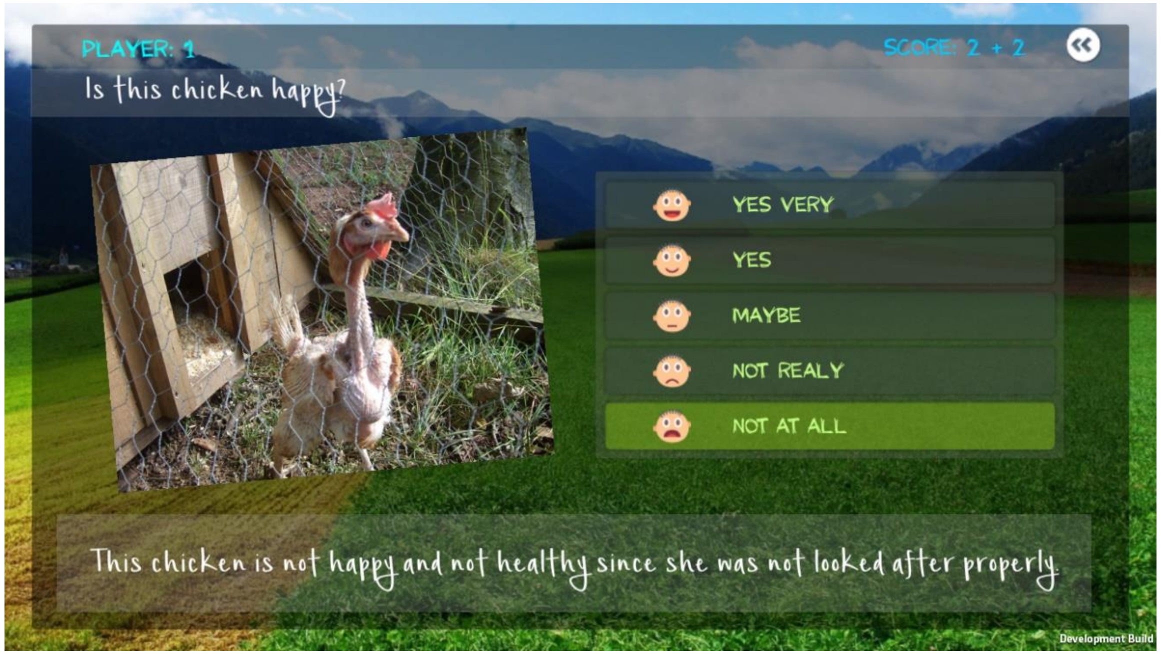

Figure S1. Example from Level 1 of Farm Animal Welfare Game.

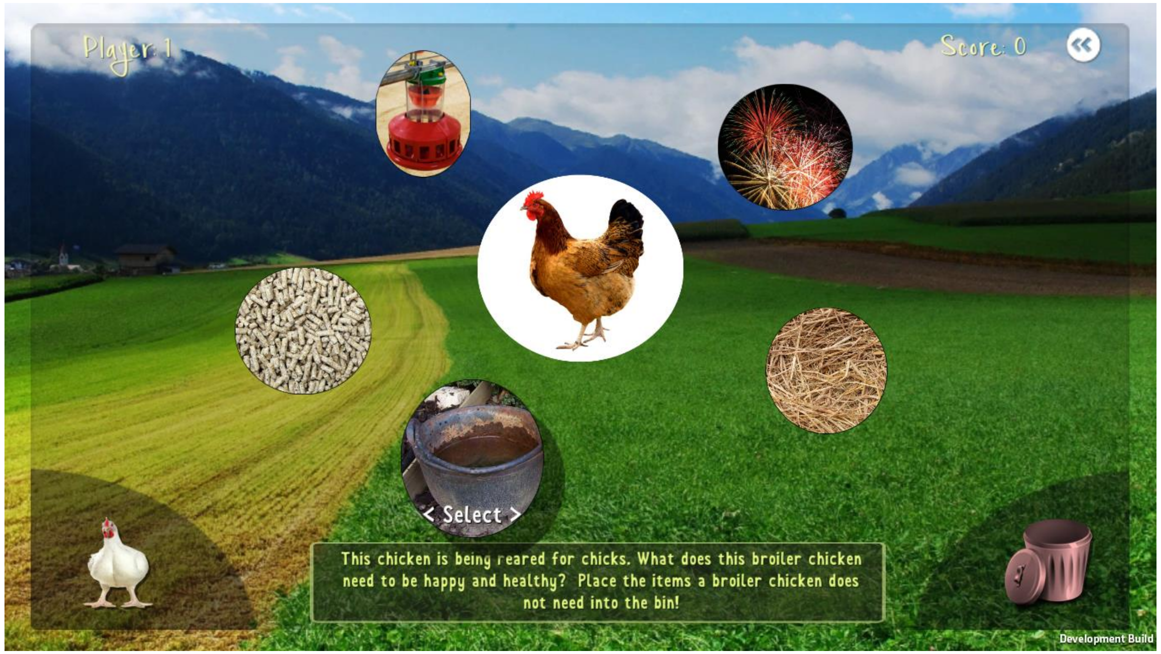

Figure S2. Example from Level 2 of Farm Animal Welfare Game.

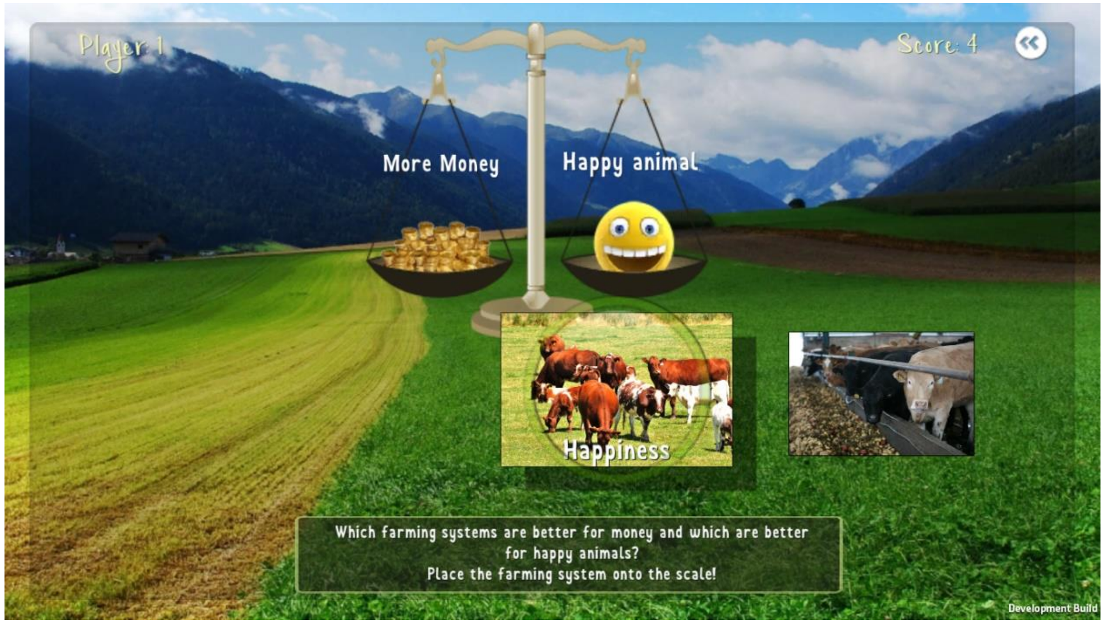

Figure S3. Example from Level 3 of Farm Animal Welfare Game.
